# Associations of children’s Big Five personality with eating behaviors

**DOI:** 10.1186/s13104-018-3768-9

**Published:** 2018-09-10

**Authors:** Margarete E. Vollrath, Svenn Torgersen, Leila Torgersen

**Affiliations:** 1 Division of Mental and Physical Health, Norwegian Institute of Public Health, PO Box 222, Skøyen, 0213 Oslo, Norway; 2 Department of Psychology, Faculty of Social Sciences, University of Oslo, PO Box 1094, Blindern, 0317 Oslo, Norway

**Keywords:** Children, Personality, Big Five personality, Five factor model, Temperament, Eating behaviors, Eating styles, Obesity, Overweight, Age, Gender

## Abstract

**Objective:**

Our aim is to examine the associations of the Big Five personality factors with eating behaviors in children using a cross-sectional study in 1543 randomly Norwegian 7–12 year olds.

**Results:**

Mothers rated the hierarchical personality inventory for children, and the child eating behaviour questionnaire to describe her child. Personality and eating behaviors were substantially associated in bivariate correlations and multivariate analyses of variance. The strongest predictors of eating behaviors were neuroticism, followed by agreeableness and conscientiousness. Neuroticism correlated the highest with slow eating, emotional undereating, food responsiveness, and emotional overeating, and showed minor associations with satiety responsiveness, and fussiness. Neuroticism was not associated with enjoyment of food. Agreeableness was associated with low fussiness, low emotional undereating, low food responsiveness and low emotional overeating, conscientiousness was associated with low satiety responsiveness, and food responsiveness, and extraversion and imagination were associated with high enjoyment of food.

## Introduction

Across the world, children’s overconsumption of unhealthy foods, and problems with reigning in their appetite in an environment where foods are easily accessible everywhere, have increasingly come into focus. Children’s eating behaviors, or appetitive traits, comprise responsiveness to internal signals (satiety), food avoidance tendencies (slow eating, fussiness), responsiveness to external food cues, use of food to regulate emotions (undereating and overeating), and enjoyment of food [1, 2].

In the quest to find broad biologically rooted character traits that may explain eating behaviors, studies have used temperamental traits, clinical eating disorders, and screening scales for children’s emotional and behavioral problems [2]. However, we argue that children’s emotional, behavioral, and cognitive dispositions must be conceptualized in terms of the Big Five personality system. This model of independent factors has been considered necessary and sufficient in personality psychology since the 1980s, has been adopted for adolescents, and is increasingly being adopted for children [3].

The Big Five personality factors are extraversion, agreeableness, conscientiousness, neuroticism, and openness to experience. Recent research shows that children’s Big Five are related their consumption of obesogenic foods and drinks, which in turn is predicted by eating behaviors [4]. Notably, the traits of neuroticism, low conscientiousness, and low agreeableness are related to an excessive consumption of sweet foods and drinks [5]. In adults, a recent study showed that neuroticism was associated with more emotional and external eating, extraversion with external eating, and conscientiousness with restrained eating and reduced external eating [6]. In children, such data is lacking—and the present study offers to address this research gap.

## Main text

### Method

#### Dataset

Our data originate from a pilot study designed to test and validate assessment well known assessment scales for children’s behavioral and emotional adjustment in Norway. Details regarding this study have been described earlier [4, 7]. Ultimately, the scales were piloted for the 8-year follow-up of the Norwegian Mother and Child Cohort Study [8]. In brief, in 2007, Statistics Norway mailed questionnaires to 4000 randomly selected households across Norway with a target child in the age range 7–12 years. The questionnaires were in Norwegian and addressed to the mothers in the households. Statistics Norway retained all addresses and delivered completely anonymous data.

#### Participants

Mothers were asked to choose the youngest of her children in the 7–12 year age-range and complete a questionnaire describing the child. This yielded 1543 complete questionnaires, corresponding to a 39% return rate (800 boys and 743 girls). The children’s mean age was 8.9 years [standard deviation (SD) 1.7 years] and mothers’ mean age was 38.2 years (SD 4.9 years). Most families were intact (86.6% of the parents were married or cohabiting with their partner). Around 50% of the mothers had completed higher education (college, university, i.e. at least 16 years of education), which corresponds well with the 49% rate of higher education for the 30–39 year female cohorts in 2007 [9].

#### Instruments

Mothers rated their child’s personality using the Norwegian hierarchical personality inventory for children (HiPIC), which bases on the flemish HiPIC, one of the first questionnaire to assess the five personality factors in children [10, 11]. The HiPIC assesses the *five personality factors of* extraversion, agreeableness, conscientiousness, neuroticism and imagination. The HiPIC is constructed hierarchically. It comprises 144 items that are assembled into 18 subtraits with 8 items each. These subtraits are summarized into five factors.

1. Extraversion, describing a sociable, talkative, energetic, and optimistic child.
2. Agreeableness, describing an altruistic, unselfish, friendly, trusting and docile child.
3. Conscientiousness, describing a well-organized, ambitious, and perseverant child.
4. Neuroticism, describing an anxious, moody, and self-conscious child.
5. Imagination (openness to experience in adults) describing a curious, creative, and quick-witted child.

The HiPIC was translated into Norwegian and validated in several samples. The Norwegian version has demonstrated excellent reliability and convergent validity [11].

Further, mothers rated their child’s eating behaviors by means of the CEBQ, a widely used questionnaire designed to capture *eating behavior in children* [12]. The CBEQ has 32 items, assembled into seven scales. Four scales express lower appetite and avoidance of foods, three further scales express greater appetite and approach toward food.

1. Satiety responsiveness (responding to inner cues of satiety).
2. Slowness in eating (dawdling with food, not finishing meal).
3. Fussiness with food (being selective about food and rejecting new foods).
4. Emotional undereating (eating *less* when upset).
5. Food responsiveness (attracted to food cues),
6. Enjoyment of food (pleasure when eating).
7. Emotional overeating (eating *more* when upset).

For each scale, we computed Cronbach’s alpha reliability estimates, which varied between *α *= 0.66 (slow eating) and *α *= 0.91 (fussiness). We included the children’s gender and age as confounders. We did not control for parental education, because education is correlated with parental personality, which in turn is genetically associated with the child’s personality. Controlling for parental education would thus eliminate a part of the genetic variance of personality shared between parents and children.

#### Statistics

Given that both the personality factors and the CEBQ eating behaviors show high inter-correlations, we computed a multivariate analysis of variance by means of the statistical package for the social sciences (SPSS), version 24 [13]. All scales were standardized before computing the model. Age was entered as a continuous variable, gender as categorical variable coded 0 for boys and 1 for girls.

### Results

Table 1 presents bivariate associations among and between all variables in this study. As discussed in a previous paper [11], some of the correlations between the Big Five personality factors were higher than desirable—the ideal being “zero” correlations, because the Big Five factors are construed as independent dimensions. It is important to note the *r *= 0.61 correlations between extraversion and imagination. The correlations between the eating styles were generally lower, but with notably high correlations of satiety responsiveness with fussiness (*r *= 0.50) and with enjoyment of food (*r *= − 0.57), as well as between food responsiveness and emotional overeating (r = 0.59). The intra-instrument correlations suggest that the scales of both the HiPIC and the CEBQ need to be adjusted for each other when examining the associations across personality and eating behaviors.

**Table 1:** Correlations between personality factors and eating behaviors

	E	A	C	N	I	SR	SE	FS	EU	FR	EF	EO	Gender	Age
Extraversion (E)	–													
Agreeableness (A)	0.24	–												
Conscientiousness (C)	0.24	0.44	–											
Neuroticism (N)	− 0.53	− 0.44	− 0.31	–										
Imagination (I)	0.61	0.27	0.52	− 0.43	–									
Satiety responsiveness (SR)	− 0.03	− 0.15	− 0.14	0.14	− 0.06	–								
Slow eating (SE)	− 0.07	− 0.07	− 0.04	0.18	− 0.05	0.24	–							
Fussiness (FS)	− 0.17	− 0.22	− 0.17	0.22	− 0.18	0.50	0.16	–						
Emotional undereating (EU)	− 0.07	− 0.19	− 0.08	0.20	− 0.04	0.34	0.16	0.16	–					
Food responsiveness (FR)	− 0.08	− 0.24	− 0.20	0.25	− 0.09	− 0.20	0.02	− 0.03	0.09	–				
Enjoyment of food (EF)	0.18	0.12	0.12	− 0.13	0.20	− 0.57	− 0.19	− 0.56	− 0.10	0.36	–			
Emotional overeating (EO)	− 0.14	− 0.23	− 0.16	0.31	− 0.12	0.01	0.10	0.07	0.36	0.59	0.15	–		
Gender (boys = 0, girls = 1)	0.05	0.02	0.07	0.02	0.07	0.04	0.03	− 0.08	0.03	0.05	0.02	0.01	–	
Age	− 0.14	0.14	− 0.01	0.02	− 0.13	− 0.18	− 0.10	− 0.14	0.01	0.09	− 0.11	0.00	0.03	–

Pearson correlations ≥ 0.09 are significant at *P* ≤ 0.001

The Five Factor factors correlated significantly with the entire range of eating behaviors. Extraverted children fussed less with their food, enjoyed food more, and overate less frequently. Agreeable children were less responsive to satiety, fussed less with their food, did not eat less when emotionally upset, were not responsive to food cues and did not overeat when upset. Conscientious children showed the same pattern of eating behaviors as agreeable children, but the correlations were smaller. Children high in neuroticism were more responsive to satiety, tended to eat slowly, ate less when emotionally upset, were more responsive to food, and overate when emotionally upset. At the same time, they enjoyed food less. Imaginative children were less fussy with food, enjoyed food more, and did not tend to overeat when upset. Gender was not associated with either personality or eating behaviors. In contrast, higher age was associated with lower extraversion, higher agreeableness, and lower imagination, less satiety responsiveness, lower fussiness, and lower enjoyment of food.

Table 2 shows a more fine-grained pattern of associations between the 18 personality subtraits of the HiPIC and the CEBQ eating behaviors. The extraversion subtrait of optimism correlated the highest with enjoyment of food, and the agreeableness subtraits of egocentrism, compliance, and irritability correlated with the highest with fussiness, food responsiveness, and emotional overeating. The conscientiousness subtraits of concentration and perseverance correlated the highest with low food responsiveness and low emotional overeating, whereas the neuroticism subtraits of anxiety and low self-confidence correlated the highest with fussiness, food responsiveness, and emotional overeating. Finally, the three imagination subtraits correlated the highest with enjoyment of food.

**Table 2:** Correlations between personality subtraits and eating behaviours

Factors	Subtraits	Satiety responsiveness	Slow eating	Fussiness	Emotional undereating	Food responsiveness	Enjoyment of food	Emotional over-eating
E	Energy	0.04	− 0.04	− 0.04	0.03	− 0.02	0.06	− 0.01
	Expressiveness	0.02	0.00	− 0.13	0.00	0.00	0.16	− 0.06
	Optimism	− 0.06	− 0.08	− 0.18	− 0.10	− 0.12	0.20	− 0.18
	Shyness	0.08	0.10	0.18	0.13	0.11	− 0.13	0.17
A	Altruism	− 0.07	− 0.05	− 0.18	− 0.07	− 0.06	0.16	− 0.08
	Dominance	0.03	− 0.04	0.02	0.05	0.10	0.00	0.04
	Egocentrism	0.17	0.11	0.25	0.19	0.27	− 0.12	0.28
	Compliance	− 0.15	− 0.05	− 0.22	− 0.17	− 0.19	0.12	− 0.17
	Irritability	0.12	0.08	0.17	0.20	0.25	− 0.07	0.27
C	Concentration	− 0.12	− 0.11	− 0.14	− 0.09	− 0.20	0.11	− 0.21
	Perseverance	− 0.16	− 0.04	− 0.18	− 0.13	− 0.25	0.09	− 0.21
	Order	− 0.14	− 0.02	− 0.16	− 0.06	− 0.18	0.10	− 0.12
	Achievement striving	− 0.02	0.02	− 0.05	0.02	− 0.01	0.09	0.00
N	Anxiety	0.13	0.16	0.20	0.20	0.25	− 0.08	0.31
	Self-confidence	− 0.12	− 0.16	− 0.20	− 0.16	− 0.20	0.15	− 0.25
I	Creativity	0.00	− 0.01	− 0.13	0.02	− 0.04	0.17	− 0.07
	Intellect	− 0.08	− 0.09	− 0.15	− 0.07	− 0.15	0.14	− 0.17
	Curiosity	− 0.07	− 0.02	− 0.17	− 0.04	− 0.03	0.19	− 0.05

*E* extraversion, *A* agreeableness, *C* conscientiousness, *N* neuroticism, *I* imagination

Pearson correlations ≥ 0.09 are significant at *P* ≤ 0.001

**Fig. 1:**
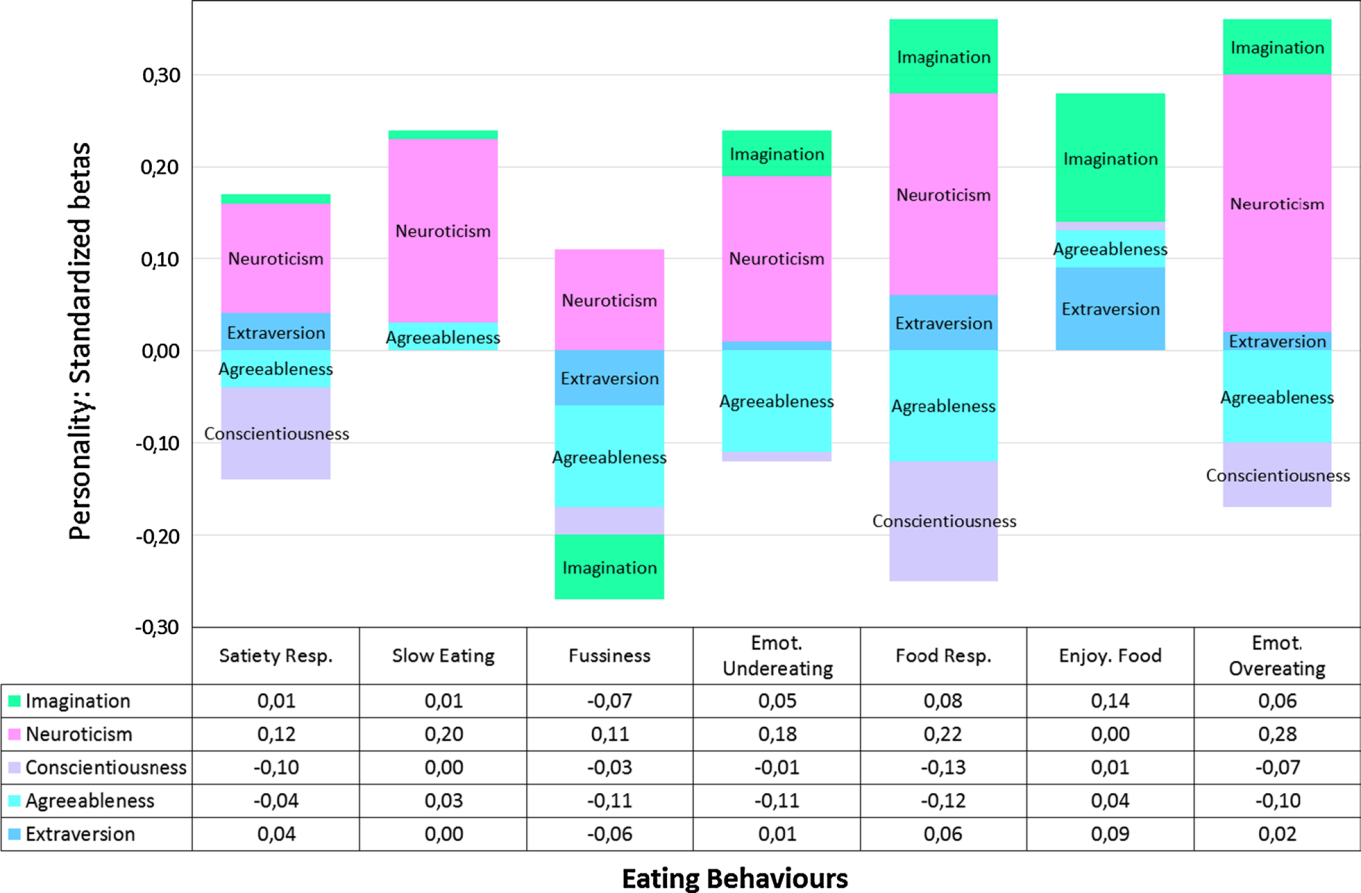
Multivariate associations between personality factors and eating behaviors in children. General linear model. Numbers in the table are standardized betas; betas ≥ 0.065 are significant at *P* ≤ 0.05. Effects of age are not presented. The independent variables (age, personality factors) are mutually adjusted; likewise, the dependent variables are mutually adjusted. The *multivariate* summarized partial coefficients for the associations of single personality factors across eating styles are: Extraversion; *F *= 2.948, *P *= 0.005, partial *η*^2^= 0.01; agreeableness; *F *= 7.297, *P *= 0.000, partial *η*^2^= 0.03; conscientiousness; *F *= 5.972, *P *= 0.000, partial *η*^2^= 0.03; neuroticism; *F *= 20.674, *P *= 0.000, *η*^2^= 0.09; imagination; *F *= 3.786, *P *= 0.000, partial *η*^2^= 0.02. Total effects of personality on eating behaviors: partial *η*^2^ = 0.178, i.e. 17.8% of the variance

The results of the multivariate analysis of variance, which takes into account the inter-correlations between personality traits as well as the inter-correlations among eating styles are presented in Fig. 1.

Neuroticism explained the lion’s share of variance across eating behaviors, correlating with both avoidance and approach eating behaviors. Agreeableness explained the second highest share of variance across eating, showing negative associations with fussiness, emotional undereating, food responsiveness, and emotional overeating. Conscientiousness ranked third, showing associations with both satiety responsiveness and food responsiveness. Imagination ranked fourth and Extraversion ranked fifth, both factors showing associations with enjoyment of food.

### Discussion

Our results make sense when considering other behaviors affected by the Big Five personality factors. In adults, neuroticism is a trait associated with unhealthy, risky and addictive behaviors, [14, 15], and more specifically and with external and emotional eating [6, 16]. In children, neuroticism is associated with higher weight and higher consumption of sweet foods and drinks [4, 5]. Agreeableness is a moderate predictor of health behaviors in adults, but in children, it shows associations with healthy food choices and lower BMI, as well as with better compliance with diabetes treatment [5, 17]. This may have to do with the salience of compliance and irritability in children’s agreeableness, subtraits that may merge into conscientiousness and neuroticism in adults. Conscientiousness is a very powerful predictor of health behaviors in adults [18], not the least because of the crucial importance of self-control for all adaptive human behaviors. In this study, the conscientiousness subtraits of order, concentration, and perseverance underline the importance of executive functioning, behavioral adaptation, and low impulsiveness for regularity in eating. Imagination explained correlated with lower fussiness and enjoyment of food, findings paralleling the healthier food choices in adults with high Openness to experience fish and more fruit and vegetables [19].

## Limitations

- The sample is not representative of the entire Norwegian population, especially because the questionnaire has not been translated into other languages.
- The study is cross-sectional study, which does not allow determining causality. Personality may be antecedent to eating behaviors or vice versa. However, the common assumption is that broader constructs, such as personality, precede more narrow constructs.
- Shared method variance between the two sets of scales and rater bias in the mothers, who filled in both sets of scales. This has certainly has inflated the correlations.
- Associations may be spurious and due to shared genes coding for both personality and eating behaviors.
- Shortcomings of the questionnaires. There were high inter-correlations between the HiPIC factors, which is a known problem when assessing the personality of children and adolescents. In a similar vein, there were high inter-correlations between the CBEQ scales. Even so, we chose to stick to the original scales of both questionnaires in order to make our findings comparable with future research.

## Abbreviations

HiPIC: hierarchical personality inventory for children
CEBQ: children’s eating behaviour questionnaire
SPSS: statistical package for the social sciences
